# Post‐COVID‐19 Exacerbation of a Stable Fibrous Dysplasia: A Case Report

**DOI:** 10.1002/ccr3.70474

**Published:** 2025-04-22

**Authors:** Mohammed Taib Fatih, Mohammed Abdalla Mahmood, Mohammed Khalid Mahmood, Amanj Ismael Tahir, Handren Ameer Kurda, Mohammed Aso Abdulghafor, Balen Hamid Qadir, Zana Fuad Noori

**Affiliations:** ^1^ Department of Dentistry Komar University of Science and Technology Sulaimani Iraq; ^2^ College of Dentistry Sulaimani University Sulaimani Iraq; ^3^ Aix‐Marseille University, CNRS, EFS, ADES Marseille France; ^4^ Dentistry College American University of Iraq‐Sulaimani AUIS Sulaimani Iraq

**Keywords:** COVID‐19, fibrous dysplasia, reactivation, SARS‐CoV‐2, tumorigenesis

## Abstract

Fibrous dysplasia (FD) is a rare, benign fibro‐osseous lesion characterized by replacement of normal bone with extensive fibrous stroma due to abnormalities in osteoblast differentiation. After puberty and during adulthood, FD lesions usually become quiescent. However, some cases show signs of regrowth and reactivation. Here, we report a previously stable maxillary FD case in a 32‐year‐old man reactivated after a mild COVID‐19 infection. We hypothesize that SARS‐CoV‐2 may utilize diverse mechanisms to induce tumor/cancer in multiple organs, including initiating inflammatory cascades and modifying tumor‐suppressing pathways. The capacity of SARS‐CoV‐2 to enhance the expression of proinflammatory and tumorigenic molecules necessitates further research to ascertain any correlation between this viral infection and FD or other similar diseases.

**Summary**

- This paper reports the reactivation of a stable case of fibrous dysplasia after COVID‐19 infection and discusses the plausible biological mechanisms that may link the two entities.

## 1 Introduction

In 1938, Lichtenstein was the first to define fibrous dysplasia (FD) as a benign fibro‐osseous disease of the bone. FD is an uncommon bone disease that is marked by aberrant fibrous tissue replacing normal bone. It is possible to distinguish between a systemic lesion (the polyostotic form) and a local form (the monostotic FD). McCune–Albright syndrome is a polyostotic FD characterized by hormone abnormalities and café‐au‐lait spots on the skin [1, 2, 3]. Compared to polyostotic FD, monostotic FD is more prevalent. FD contributes to about 7% of all benign bone tumors and 2.5% of all bone lesions. It is challenging to ascertain the incidence of FD since many individuals are asymptomatic and are found by chance following radiographic imaging [4, 5].

The hallmark of craniofacial FD is usually a slowly expanding mass that can be painful and result in facial asymmetry. In the craniofacial region, the maxilla is most frequently impacted, followed by the mandible and the frontal bone [6]. Even though 90% of the lesions are monostotic, meaning they only impact one bone, this is only true for the mandible in the craniofacial region since FD lesions in the maxilla can affect many bones by crossing sutures into the sphenoid, zygoma, base of the skull, and frontonasal bones [7, 8]. The posterior portion of the maxilla and mandible is where most of the unilateral lesions are found [9]. The degree of impairment is determined by the existence and severity of symptoms. In particular, the effects on the quality of life have not yet been evaluated.

FD typically manifests in the first or second decade of life and begins in infancy or early adolescence. Usually, FD lesions of the jaws manifest in the second or third decade of life [10].

The differential diagnosis of craniofacial FD includes ossifying fibroma, chronic diffuse sclerosing osteomyelitis of the mandible, cherubism in children, osteoblastoma, and central giant‐cell granuloma mimicking fibro‐osseous lesions [1, 11].

After puberty and during adulthood, FD lesions usually become quiescent. However, some cases show signs of regrowth and reactivation. The exact causes of this exacerbation are not fully known. Nevertheless, some conditions like pregnancy and menopause are proposed, indicating the potential role of hormonal changes in the pathogenesis [10, 12, 13].

## 2 Case Presentation

A 32‐year‐old male who was previously diagnosed as having FD in the left side of the maxilla presented to the maxillofacial department of the Shar Teaching Hospital, Sulaimani, Kurdistan Region of Iraq, with concerns about rapid progression and enlargement of a previously diagnosed lesion, notably after a mild COVID‐19 infection.

**FIGURE 1:**
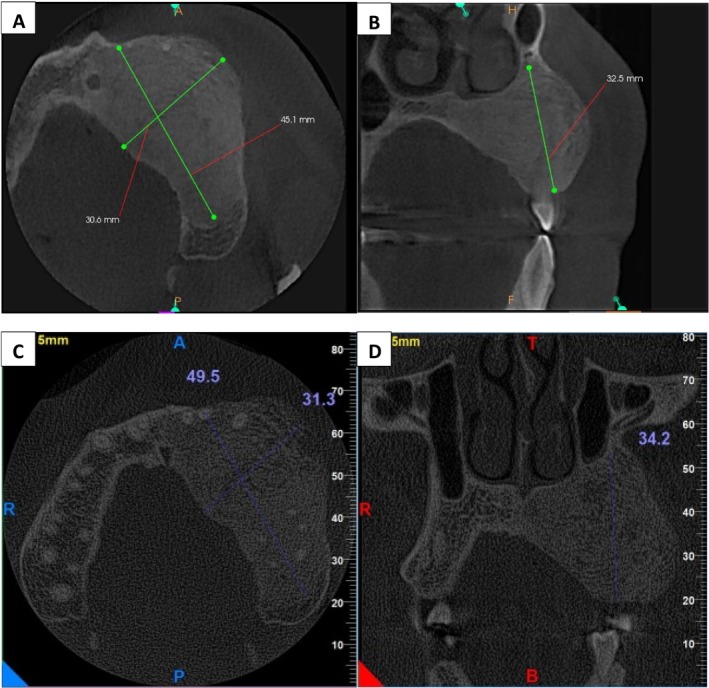
CT scan of the lesion before COVID‐19 infection in two intervals. (A, B) At the time of diagnosis. (C, D) Two years after the diagnosis.

**FIGURE 2:**
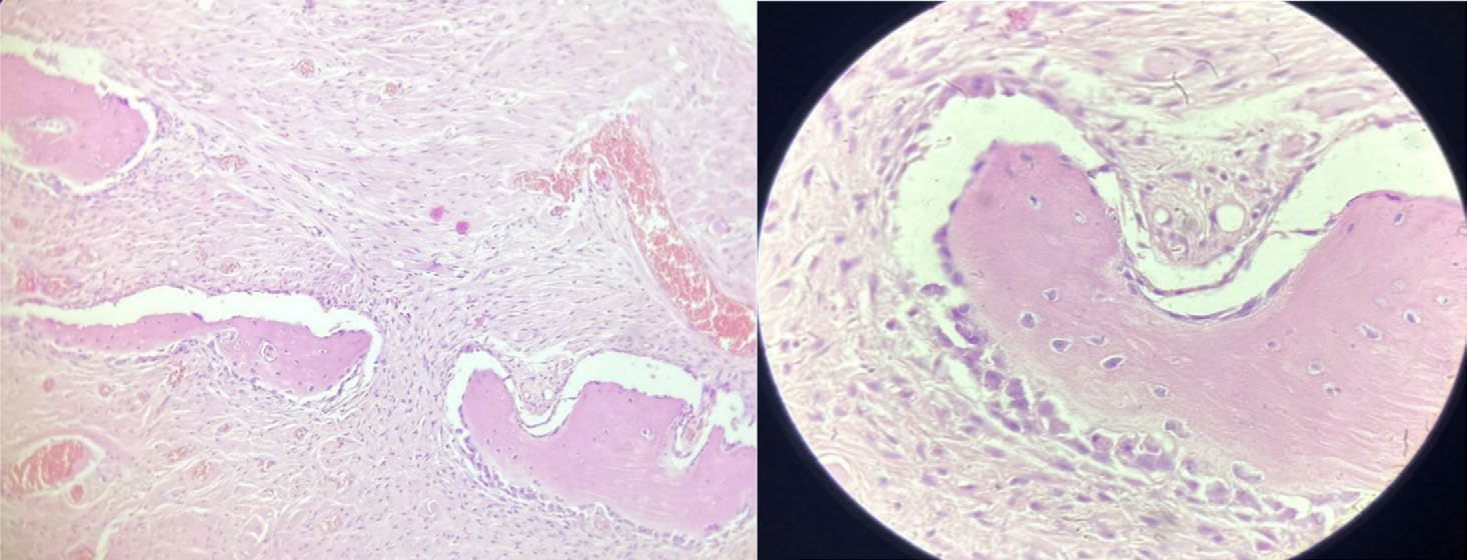
Histopathology of the lesion showing cellular fibrous tissue separated by bony trabeculae.

Back in 2015 (27 years old at that time), the patient was presented to the same hospital with a swelling in the upper jaw for a duration of 2 months A CT scan conducted in November 2015 revealed a well‐defined radiopaque mass measuring approximately 32.5 mm (superior–inferior), 30.6 mm (medio‐lateral), and 45.1 mm (anterior–posterior) (Figure 1A,B). An incisional biopsy confirmed the diagnosis of monostotic FD in the left maxilla. Figure 2 shows the histopathology of the lesion. From 2017 onwards, the patient was monitored by regular CT scans. Radiological information showed that the lesion was stable and devoid of significant growth. A follow‐up CT taken in January 2017, with dimensions measuring approximately 34.2, 31.3, and 49.5 mm, respectively, indicated very slight growth but overall confirmed stability (Figure 1C,D). Afterwards, until the onset of COVID‐19, no significant changes in the lesion were observed.

**FIGURE 3:**
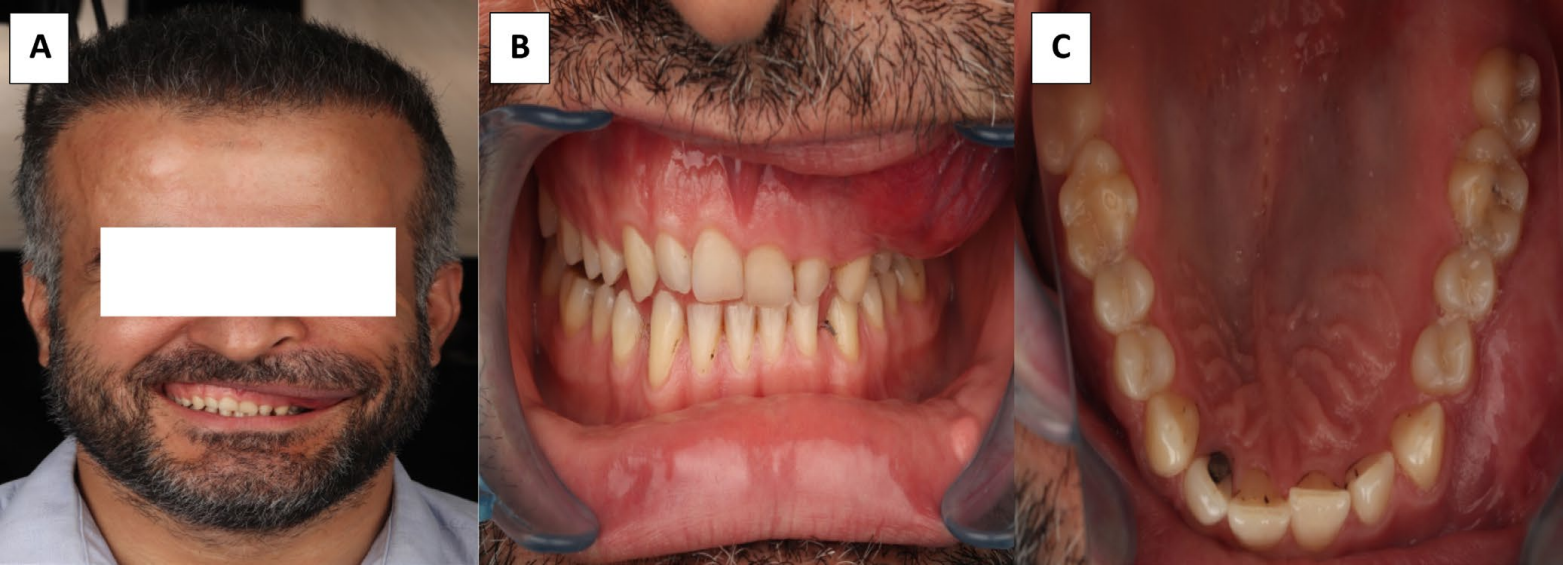
(A) Facial profile of the patient with clear asymmetry and disfigurement. (B) Intra‐oral frontal view of the lesion in the left side of the maxilla. (C) Intra‐oral occlusal view of the lesion filling the buccal vestibule, while no swelling is observed palatally.

However, in November 2020, just 3 months after a mild COVID‐19 infection, he was returned with a larger lesion. The patient reported a notable growth of the lesion after the infection. Upon clinical examination, the mass was hard, non‐movable, non‐tender to palpation; the overlying facial skin was intact with a normal color. During palpation, the swelling was felt in the buccal vestibule, starting from the left incisor and reaching posteriorly until the maxillary tuberosity. Palatally, no swelling was observed. The involving teeth were normal and vital, devoid of mobility, displacement, and pain on percussion. From the extra‐oral view, the lesion caused some detectable swelling. The lesion presented a clear disfigurement and facial asymmetry. The patient complained about not being able to occlude his teeth on the affected side and reported some interference of the lesion with occlusion, eating, and speaking. The right side of the maxilla, the mandible, tongue, and generally the oral mucosa were normal. The temporomandibular joint and regional lymph nodes were normal upon clinical examination (Figure 3).

## 3 Methods

Serological investigations including serum calcium, serum phosphorus, and alkaline phosphatase were within the normal range.

**FIGURE 4:**
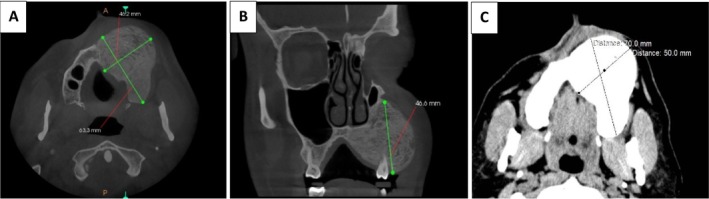
CT of the lesion after COVID‐19 infection in two intervals. (A, B) One year after the infection. (C) Two years after the infection.

**FIGURE 5:**
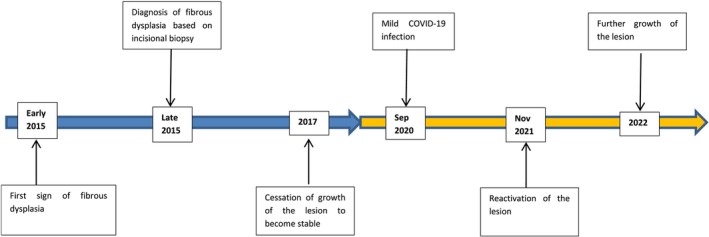
Timeline of the lesion growth.

CT imaging on April 2021 showed the lesion dimensions had increased to approximately 46.6 mm (superior–anterior), 46.2 mm (medio‐lateral), and 63.3 mm (anterior–posterior), reflecting a significant expansion after the COVID‐19 infection. The radiographic examination revealed an ill‐demarcated mixed opaque‐lucent lesion involving the left side of the maxillary alveolar process, including both buccal and palatal cortices. The entity extended from the left central incisor to the maxillary tuberosity posteriorly. Cortical bone was expanded with thinning of the cortices but without destruction. The internal structure of the lesion was homogeneous, sclerotic, and showed an abnormal bony trabecular pattern, with obliterated medullary part and absence of normal bone trabeculae. No teeth displacement and root resorption were seen, but the lamina dura of the involving teeth was absent. The floor of the associated maxillary sinus was intact with localized mild mucosal thickening (Figure 4A,B). A subsequent CT scan on May 2022 confirmed further lesion expansion, with dimensions reaching 50 mm (medio‐lateral) and 70 mm (anterior–posterior), indicating a continuing growth (Figure 4C). Figure 5 shows the timeline of the lesion growth. Table 1 shows the comparison of the lesion size before and after the COVID‐19 infection.

**TABLE 1:** Comparison of the lesion size before and after COVID‐19 infection.

Date of the imaging	Size of the radiopacity in mm (approximately)			Images
	Superior–inferior	Medio‐lateral	Anterior–posterior	
Before COVID‐19 infection				
November 2015	32.5	30.6	45.1	Figure 1A,B
January 2017	34.2	31.3	49.5	Figure 1C,D
After COVID‐19 infection				
April 2021	46.6	46.2	63.3	Figure 3A,B
May 2022	46.7	50	70.0	Figure 3C

## 4 Treatment and Outcome

In August 2023, the patient underwent left hemi‐maxillectomy. This was followed by a reconstruction surgery in June 2024 by transplanting a free soft tissue and skin flap from the anterior thigh. The patient is fit now and has returned to his nearly normal occlusion, function, and esthetic.

## 5 Discussion

FD is a hereditary disorder brought on by a somatic activating mutation in the Gsα subunit of the G protein‐coupled receptor, which causes cyclic adenosine monophosphate (cAMP) to be upregulated. This results in aberrant bone formation in an extensive fibrous stroma due to abnormalities in osteoblast differentiation. Additionally, mutant stromal fibrous dysplastic cells that cause osteoclastic bone resorption release more interleukin (IL)‐6 [14].

Age‐related alterations in radiography, histology, and clinical manifestations are characteristics of FD lesions. Due to numerous inhibitory factors that change the balance of transformed to normal cells towards the predominance of normal cells, the burden of mutated cells in FD often decreases with age, causing FD to be arrested [15]. In FD specimens from elderly people, Kuznetsov et al. found high levels of apoptosis and a decrease in the number of mutant bone marrow mesenchymal stem cells (BMSCs). Older patients' lesions also showed fewer histologic characteristics typical of FD, and in several instances, they seemed to show normal bone and marrow histology, including hematopoiesis restoration [16]. This points to a paradigm in which coexisting normal skeletal stem cells survive and allow the creation of normal marrow structures, but mutant cells in the population eventually fail to self‐renew and are eventually killed by apoptosis [4].

Clinically, FD can be categorized as aggressive (rapid growth ± discomfort, pathologic fractures, malignant changes, etc.), quiescent (stable), or nonaggressive (slow growing). As patients approach adolescence, the course of FD frequently slows down [5, 12].

In adulthood, certain cases of FD may reactivate. The etiology of this aggravation remains incompletely understood. Nevertheless, certain risk factors have been suggested. The reactivation of FD cases after pregnancy and menopause has been recorded, highlighting the importance of hormones in the pathogenesis. Additional potentially relevant factors, such as the use of traditional medicine and the gradual decrease in water fluoride levels, have also been documented [10]. Dysplastic bone generally does not undergo significant remodeling to the normal lamellar type, rendering it potentially susceptible to stimuli that may reawaken it [17].

There exists a rich literature on the oncogenic capacity and tumorigenesis of viruses [18, 19]. SARS‐CoV‐2 and its COVID‐19 infection have been studied mainly in relation to its respiratory effects and eventual mortality [20]. However, its long‐term impacts are still under investigation.

To our knowledge, this is the first reported FD case that showed reactivation after infection with SARS‐CoV‐2. However, a similar pattern of exacerbation after COVID‐19 infection was documented in other lesions that can be compared to our case.

Grgurevic et al. documented a case of fibrodysplasia ossificans progressiva (FOP) which is a rare genetic disorder marked by intermittent episodes of heterotopic ossification (HO). This disorder involves the development of ectopic bone in muscle and soft tissues, which may be attributed to hereditary or non‐genetic factors. The typical manifestation of its non‐genetic form occurs in young people with a distinct history of localized trauma, surgical intervention, or extended immobilization following spinal cord and traumatic brain injuries. The patient was a 45‐year‐old female presenting with discomfort, swelling, and a thickening sensation in the lower abdomen and left side of the neck. The investigation of cytokines in a plasma sample collected during a flare‐up following COVID‐19 infection revealed significantly higher pro‐inflammatory cytokines compared to a flare‐up panel obtained prior to the infection. Out of the 23 cytokine levels examined, 21 exceeded normal limits [21]. Apart from reactivation after COVID‐19, another common point of this case with ours is that both exacerbations were followed by mild viral infection without the need for hospitalization.

Brance et al. reported another case of HO in a 55‐year‐old seemingly healthy man who experienced a severe SARS‐CoV‐2 infection. The substantial and progressive HO was observed around the shoulders, elbows, hips, knees, and ankles [22].

Gardini et al. documented a case of disseminated Kaposi sarcoma (KS) in a 61‐year‐old immunocompetent male following hospitalization for COVID‐19. The authors proposed that IL‐6 activity and steroid‐induced immunosuppression may have significantly contributed to the onset of KS [23].

Leis et al. reported three cases of malignant melanoma (MM) linked to severe COVID‐19. The authors proposed that SARS‐CoV‐2 elicited inflammatory tumorigenic proteins in the tumor microenvironment, perhaps contributing to de novo tumor creation in the first case, aggressive proliferation in the second, or recurrence in the third. Furthermore, elevated levels of the proinflammatory proteins present in the “cytokine storm” linked to COVID‐19, such as TNF‐α, IL‐1α, IL‐1β, IL‐6, and ferritin, may have also caused skin depigmentation or hypopigmentation, ultimately resulting in melanoma [24].

The exact biological mechanism that may link COVID‐19 infection to the reactivation of tumors and FD in our case is still to be investigated. Like several oncogenic viruses, it is suggested that SARS‐CoV‐2 may utilize diverse mechanisms to induce cancer in multiple organs. This encompasses utilizing the renin–angiotensin system, modifying tumor‐suppressing pathways through its nonstructural proteins, and initiating inflammatory cascades by increasing cytokine production, resulting in a “cytokine storm” that facilitates the emergence of cancer stem cells in target organs. Given that SARS‐CoV‐2 infection affects various organs both directly and indirectly, it is anticipated that cancer stem cells may emerge in multiple organs [18, 25].

Concerning the lack of other published FD cases despite the fact that COVID‐19 was a global pandemic with the possible infection among thousands of FD cases might be explained by the role of individual genetic susceptibility factors.

In conclusion, although the reactivation of this case right after COVID‐19 could be a mere coincidence, the capacity of SARS‐CoV‐2 to enhance the expression of proinflammatory and tumorigenic molecules necessitates further research to ascertain any correlation between these disease processes, as well as the specific implications for patients with FD or other tumors/cancers who have contracted or will develop COVID‐19 infection in the future.

## Author Contributions

**Mohammed Taib Fatih:** conceptualization, data curation, visualization. **Mohammed Abdalla Mahmood:** investigation, methodology, resources, validation, visualization. **Mohammed Khalid Mahmood:** conceptualization, methodology, project administration, writing – original draft, writing – review and editing. **Amanj Ismael Tahir:** data curation, investigation, software, validation, visualization. **Handren Ameer Kurda:** conceptualization, data curation, formal analysis. **Mohammed Aso Abdulghafor:** investigation, methodology, resources. **Balen Hamid Qadir:** conceptualization, data curation, resources. **Zana Fuad Noori:** investigation, methodology, visualization.

## Consent

Written informed consent was obtained from the patient to publish this report in accordance with the journal's patient consent policy.

## Conflicts of Interest

The authors declare no conflicts of interest.

## Data Availability

The data that support the findings of this study are available on request from the corresponding author.
